# *Bacillus anthracis* in South Africa, 1975–2013: are some lineages vanishing?

**DOI:** 10.1186/s12864-024-10631-5

**Published:** 2024-07-30

**Authors:** Kgaugelo Edward Lekota, Ayesha Hassim, Maphuti Betty Ledwaba, Barbara A. Glover, Edgar. H. Dekker, Louis Ockert van Schalkwyk, Jennifer Rossouw, Wolfgang Beyer, Gilles Vergnaud, Henriette van Heerden

**Affiliations:** 1 Faculty of Veterinary Science, Department of Veterinary Tropical diseases, University of Pretoria, Onderstepoort, 0110 South Africa; 2 Unit for Environmental Sciences and Management: Microbiology, North-West University, Potchefstroom campus, Private Bag X6001, Potchefstroom, 2520 South Africa; 3 Department of Agriculture Land Reform and Rural Development, Office of the State Veterinarian, Skukuza, Mpumalanga, 1350 South Africa; 4 Centre for Emerging Zoonotic and Parasitic Diseases, National Institute for Communicable Diseases a Division of the National Health Laboratory Services, Johannesburg, South Africa; 5 Institute of Environmental and Animal Hygiene, University of Hohenheim, Stuttgart, Germany; 6 CEA, CNRS, Institute for Integrative Biology of the Cell (I2BC), Université Paris-Saclay, Gif-sur-Yvette, 91198 France; 7 Department of Agriculture & Animal Health, College of Agriculture & Environmental Sciences, University of South Africa, 28 Pioneer Street, Florida Park, Roodepoort, 1710 South Africa

**Keywords:** *Bacillus anthracis*, Whole genome sequencing (WGS), Whole genome single nucleotide polymorphisms (wgSNP), Multi-loci variable number of tandem repeat (VNTR) assay MLVA-31

## Abstract

The anthrax-causing bacterium *Bacillus anthracis* comprises the genetic clades A, B, and C. In the northernmost part (Pafuri) of Kruger National Park (KNP), South Africa, both the common A and rare B strains clades occur. The B clade strains were reported to be dominant in Pafuri before 1991, while A clade strains occurred towards the central parts of KNP. The prevalence of B clade strains is currently much lower as only A clade strains have been isolated from 1992 onwards in KNP. In this study 319 *B. anthracis* strains were characterized with 31-loci multiple-locus variable-number tandem repeat analysis (MLVA-31). B clade strains from soil (*n* = 9) and a *Tragelaphus strepsiceros* carcass (*n* = 1) were further characterised by whole genome sequencing and compared to publicly available genomes. The KNP strains clustered in the B clade before 1991 into two dominant genotypes. South African strains cluster into a dominant genotype A.Br.005/006 consisting of KNP as well as the other anthrax endemic region, Northern Cape Province (NCP), South Africa. A few A.Br.001/002 strains from both endemic areas were also identified. Subclade A.Br.101 belonging to the A.Br.Aust94 lineage was reported in the NCP. The B-clade strains seems to be vanishing, while outbreaks in South Africa are caused mainly by the A.Br.005/006 genotypes as well as a few minor clades such as A.Br.001/002 and A.Br.101 present in NCP. This work confirmed the existence of the rare and vanishing B-clade strains that group in B.Br.001 branch with KrugerB and A0991 KNP strains.

**Supplementary Information:**

The online version contains supplementary material available at 10.1186/s12864-024-10631-5.

## Introduction

*Bacillus anthracis* is the soil-borne, spore-forming bacterium responsible for the zoonotic disease anthrax. The population structure of *B. anthracis* comprises three major clades A, B and C [1–3]. *B. anthracis* from the A clade has a global distribution while the rare B and C clades occur in more specific geographic areas [4, 5]. Anthrax outbreaks have been well documented in the Kruger National Park (KNP), South Africa since the 1920s [6]. Disease surveillance in KNP demonstrates that the epidemiological distribution of anthrax is commonly found in both northern Kruger (endemic region) with episodic enzootics recorded towards the central KNP [7]. *B. anthracis* strains isolated from anthrax outbreaks in KNP are from the A-clade and the rare B-clade. During 1975, an anthrax outbreak claimed the lives of many wild animals in the Pafuri region within northern Kruger [8]. Prior to the 1980’s, wildlife was equally affected by A- and B-clade strains [8]. The B-clade strains called Kruger B were prominent in the endemic northernmost pans in the Pafuri area before the 1990s, while ancient A-clade strains occurred in the most northern tip region along the Limpopo River southward up until central KNP and are currently the most dominant strains in this region [8]. The suppression of the Kruger B-clade occurred during the 1980–1990 as Smith et al. [6] characterized *B. anthracis* strains from 1970 to 1981 with equal A- and Kruger B-clades from the outbreaks while in 1982–1997 most were caused by A-clades and only one B-clade strain was isolated. Another B-clade strain was isolated from cheetah (*Acinoyx jubatus*) in a private game reserve outside KNP in 2011 [5].

The geographic distribution of *B. anthracis* in KNP was initially characterized using the eight-loci multiple-locus variable-number tandem repeat analysis (MLVA-8) which identified both the A and B (Kruger B branch) clades in this region [8]. The molecular typing method differentiated the major genotypes of *B. anthracis* and highlighted that in recent decades the A-clade is commonly distributed in comparison to the unique and rarely reported B-clade strains [8]. The A-clade indicated as A3 cluster is the prevailing genotype present in KNP as determined using MLVA-8 [6]. Later the inclusion of additional VNTR markers allowed to define the MLVA-25 [9] and MLVA-31 assays [10, 11] with an increased resolution. Aα (A1-A4) and Aβ could be resolved.

The canonical single nucleotide polymorphisms (canSNPs) were developed by taking advantage of the whole genome sequencing of representative strains as identified by MLVA analysis. The assay is often used in conjunction with the MLVA assay to genotype *B. anthracis* strains [3, 12]. Thirteen canSNP were initially identified that define the major clonal lineages within clades A, B and C [2, 3, 12]. The A3 cluster includes for instance ABr.001/002 (Ames/Sterne), and A.Br.Aust94 lineages. Aβ is now called Ancient A or A.Br.005/006 [12, 13]. Melt analysis of mismatch amplification mutation (melt-MAMA) is often used to run the canSNP assay [14]. The phylogenetic relationship using canSNPs is limited by the strains used to design the SNP markers including global *B. anthracis* strains with limited South African representative strains [15]. Whole genome SNP (wgSNP) analysis has a high-resolution discriminating power compared to other genotyping assays, and most importantly, has much higher phylogenetic value [16, 17]. WgSNP analysis can determine the evolution or distribution of epizootics and establish the phylogeny of minor sub-clades owing to the strictly clonal evolution of *B. anthracis* [18, 19]. Thus, the use of whole genome sequencing has enabled tracing of *B. anthracis* strains globally [18, 20]. South Africa has been hypothesized as the origin of anthrax [8] as there are high number of genetic lineages that include A.Br.Aust94 (represented by the monophyletic A.Br.101), A.Br.001/002 (Ames/Sterne), A.Br.005/006 from Northern Cape Province [15] with latter two also occurring in KNP as well as Kruger B (B.Br.001/002) [11] and B.Br.010 (unique subclade from B.Br001/002 (Kruger B) from private game reserve in Limpopo Province [7]. The B clade in KNP and A.Br.101 lineage in NCP regions are distinct compared to other SNP lineages worldwide, together with common A.Br.005/006 and A.Br.001/002 (Sterne) subclades show the diversity of strains present in South Africa. However current knowledge of *B. anthracis* diversity worldwide has allowed to convincingly establish that the presence of A.Br.Austr94, A.Br.001/002 (Ames/Sterne), as well as the B-clade is the result of secondary, reintroduction events, and not the reflection of an ancestral diversity [21]. As *B. anthracis* is regarded as a monomorphic pathogen, MLVA and wgSNP analysis is the ideal approach for genotyping. In this study, 319 strains from South Africa were characterized with MLVA-31 and ten B-clade strains were whole-genome sequenced. We further compared the B-clade South African sequenced genomes with publicly available genomes that reflect the canonical lineages of *B. anthracis* globally to investigate the genetic diversity and transmission patterns of the anthrax outbreaks.

## Methods

### Collection of *Bacillus anthracis* strains

Strains spanning outbreaks from 1970 to 2013 from endemic regions in South Africa, namely the KNP and Northern Cape Province (NCP) were used in this study. A total of 319 *B. anthracis* strains were obtained from the culture collections of the National Institute for Communicable Diseases (NICD), the Onderstepoort Veterinary Institute (OVI reference laboratory), and the University of Pretoria (Supplementary Table 1). This study used B-clade strains (*n* = 10) isolated in 1975 from soil and animals in Pafuri, KNP. The strains were genotyped using whole-genome sequencing (Table 1). *B. anthracis* DNA (*n* = 319) which included the ten sequenced strains was characterised with MLVA-31 (Supplementary Table 1). All the strains originated from the Skukuza State Veterinary Services, which identified the strains using OIE-recommended microbiology and molecular techniques. Sensitivity to penicillin and gamma-phage, haemolysis and motility tests were determined on the pure cultures for identification of *B. anthracis* as described previously [15] .

**Table 1:** Description of *Bacillus anthracis* B-clade strains from Pafuri in Kruger National Park in South Africa analyzed using whole genome sequencing in this study

Strain ID	Location and Source	Year	SNP group
A3	Pafuri, Soil	1975	B.Br.001
A5	Pafuri, Soil	1975	B.Br.001
A8	Pafuri, Soil	1975	B.Br.001
A11	Pafuri, Soil	1975	B.Br.001
A16	Pafuri, Soil	1975	B.Br.001
A19	Pafuri, Soil	1975	B.Br.001
C13	Pafuri, Soil	1975	B.Br.001
HP8	Pafuri, Soil	1975	B.Br.001
HP12	Pafuri, Soil	1975	B.Br.001
Z21	Pafuri, Kudu	1975	B.Br.001

### DNA extractions and PCR

DNA extraction was carried out using the DNA Blood Mini Kit (Qiagen) according to the manufacturer’s instructions for Gram-positive bacteria with 20 mg/mL lysozyme (Sigma Aldrich). The DNA was quantified with the Qubit Fluorometer (Invitrogen™) using the broad range assay according to the manufacturer instructions. The quality of the extracted DNA was visualized under UV light after electrophoresis on 0.8% agarose gel electrophoresis and ethidium bromide staining. PCR confirmation of *B. anthracis* strains was performed as previously described [22].

### Multiple locus variable-number tandem repeat analysis-31

DNA was subjected to PCR, targeting 31 VNTR markers with fluorescently labelled forward primers, multiplexed in seven reactions as described by Beyer et al. [10]. Multiplex PCRs were prepared in 15 µl volumes, consisting of 1x MyTaq HS Mix (Bioline), 0.4–1 µM primers, and 5 ng of template DNA. The PCR conditions for multiplexes 1 to 4 and 7 were as follows: initial denaturation at 95 °C for 2 min; 35 cycles of denaturation at 95 °C for 20 s; annealing at 60 °C for 30 s; elongation at 72 °C for 2 min; followed by a final elongation step at 72 °C for 5 min. The PCR conditions for multiplexes 5 and 6 were as follows: initial denaturation at 95 °C for 2 min; 30 cycles of denaturation at 95 °C for 20 s; annealing at 50 °C for 30 s; elongation at 72 °C for 2 min; followed by a final elongation step at 72 °C for 5 min. Amplicons were separated using an ABI 3500xl Genetic Analyzer (Applied Biosystems), a G5 filter set, and a 51 cm POP-7 capillary.

The size of the resultant amplicon fragments was determined using GeneMapper ID-X© software (Applied Biosystems) with the internal LIZ1200© size standard (Applied Biosystems). Fragment size normalisation was performed by including three *B. anthracis* strains, Sterne 34F2 vaccine strain, Vollum (A70), and Ames (A93) as controls. Base-pair sizes for each allele were converted into copy numbers using a previously described algorithm [9, 10], with the copy code convention proposed by Thierry et al. [23] (Supplementary Table 2). The datasets of archival strains from 1975 to 2013 (see Supplementary Tables 1 and 2) were validated by inter-laboratory comparisons between the Department of Veterinary Tropical Diseases, University of Pretoria, and the Special Bacterial Pathogens Reference Laboratory, National Institute for Communicable Diseases, and by independent genotyping of the strains library. Inter-laboratory comparison data for amplicon size calling and copy number is represented in Supplementary Table 2. The datasets of archival strains from 1975 to 2013 (*n* = 319), includes B-clade strains as well as strains from other southern African countries. Genetic distance and cluster analysis were performed using the unweighted pair group method using arithmetic mean (UPGMA), and minimum spanning analysis was performed using categorical data in BioNumerics© version 6.4.4 (Applied-Maths, Belgium). Due to some unusual product sizes, the amplicons of selected strains for Bams23, vrrA, and Bams30 were sequenced in order to verify the repeat units.

### High-throughput sequencing and read mapping analysis

The extracted *B. anthracis* DNA (*n* = 10) was subjected to library preparation using the Nextera XT DNA Sample Prep Kit (Illumina) protocol. A sequence-paired end library was performed on the Illumina HiSeq 2500 (Illumina). The quality of the genome-sequenced reads was assessed using FastQC software 0:10.1 [24]. Trimommatic version 0.33 [20] was used to remove the sequenced adapter and ambiguous nucleotide reads. The trimmed reads of the *B. anthracis* sequences were mapped to the *B. anthracis* Ames ancestor (Accession: NC_007530.2) using the Burrows-Wheeler Aligner version 0.7.12 [21].

### *De novo* assembly, annotation and virulence genes detection

Trimmed reads were *de novo* assembled using the Shovill Faster SPAdes v1.1.0 pipeline (Seemann, 2017). QUAST v. 4.5 [14] was used to assess the quality of the associated assembled genome using default parameters. CheckM [15] was additionally used to assess the potential contaminants in each assembled genome by comparison with the *B. anthracis* Ames ancestor using default parameters. BLASTn [16] was used to align assembled contigs with the *B. anthracis* Ames ancestor as a reference. For consistency and the removal of contaminants, multiple genome alignments were built using the progressive MAUVE tool [17]. Feature prediction and annotation of the sequenced genomes were performed using the NCBI prokaryotic genome annotation pipeline (PGAP) (https://www.ncbi.nlm.nih.gov/genome/annotation_prok/) and rapid annotation RAST [18]. The AMRFinderPlus [19] was used to determine the virulence genes of the sequenced strains.

### Whole genome SNP analysis

Whole genome SNP analysis was conducted on the B-clade strains (*n* = 10) (Table 1) and publicly available genomes previously described in Lekota et al. [5, 15]. SAMTools version 1.3 [17] was used to generate in silico overlapping 200 bp reads from complete and draft genome assemblies retrieved from GenBank. The nucleotide accession numbers of the ten sequenced strains are listed in Supplementary Table 3. The sequence reads of the strains and retrieved genomes were aligned to the *B. anthracis* Ames ancestor reference chromosome using the Burrows-Wheeler Aligner version 0.7.12 [18]. The unified genotyper method in GATK version 3.7 [19] was used to call for SNPs as previously described [20]. SNP positioning sets of all the sequenced and compared genomes were deduced from the aligned genomes of the *B. anthracis* Ames ancestor. SNPs identified in genomic regions missing at one or more strains were excluded from the selection of aligned informative SNP sites. SNPs with parsimony-informative sites that were present in all genome sequences were used for phylogenetic analysis. A phylogenetic tree of the whole genome’s filtered SNPs was constructed using the molecular evolutionary genetics analysis tool version 7 [21]. The trees were generated using the maximum likelihood method with a bootstrap replication value of 500 or the maximum parsimony method implemented in BioNumerics (Applied-Maths, Belgium).

## Results

### Microbiological and molecular tests

The strains under investigation in this study were confirmed as *B. anthracis* based on colony morphology, microscopy, and classic diagnostic tests. All strains were observed to be non-motile Gram-positive bacilli with square ends, non-haemolytic on blood agar, and sensitive to penicillin and gamma-phage (lysis). Real-time PCR was used to confirm identification of all the *B. anthracis* strains by targeting *B. anthracis* protective antigen (BAPA), Spore-associated surface protein (SASP), and *capC* regions [25, 26].

### MLVA with 31 loci

**Fig. 1:**
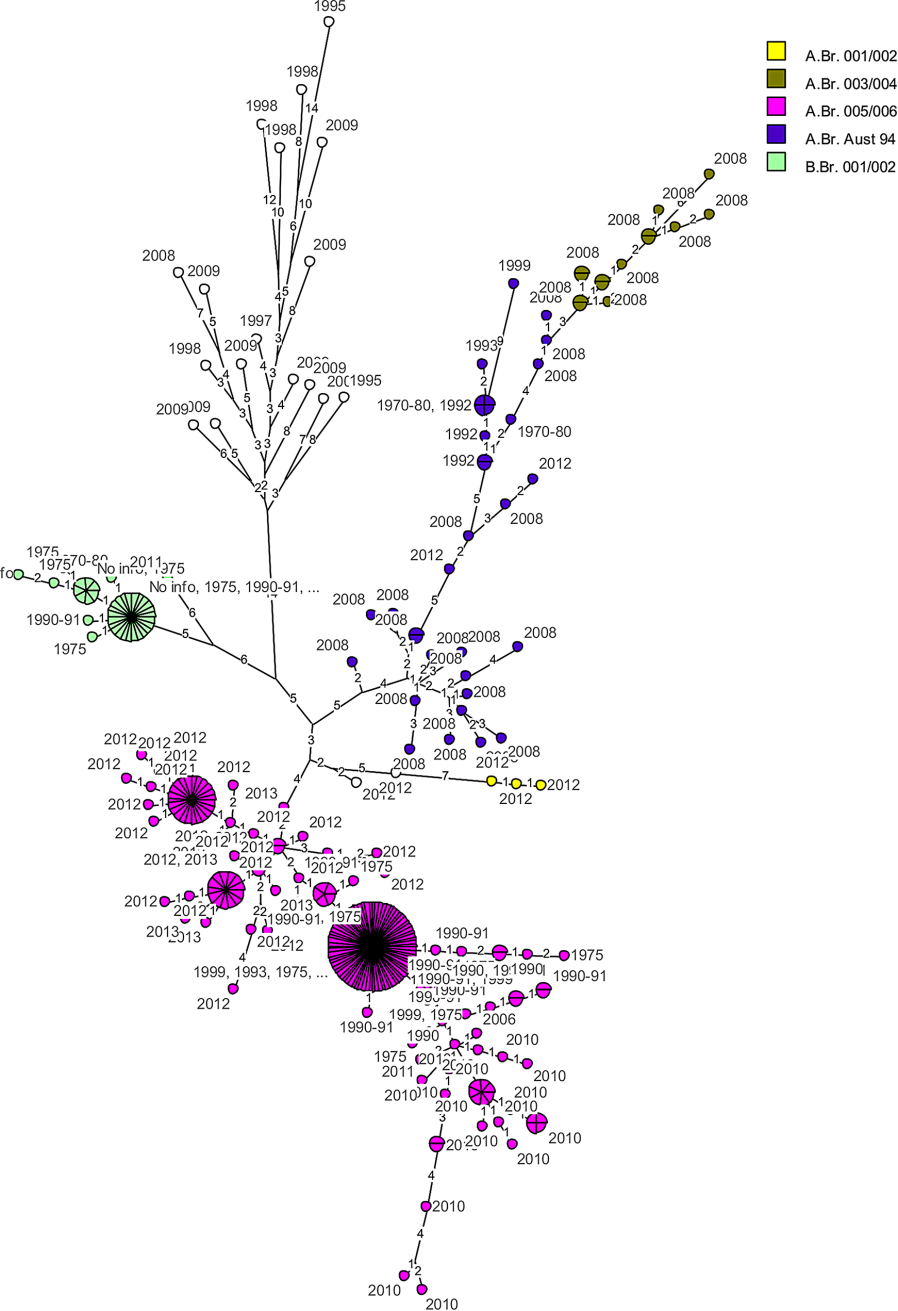
Minimum spanning trees based on categorical coefficient data of 31 VNTR loci for *Bacillus anthracis* MLVA fingerprints generated in BioNumerics (version 6.6). The MST tree is arranged visually according to 1 A: the year of the anthrax outbreak, designated by colour. 1B: the region of the anthrax outbreak, designated by colour and indicating SNP lineages

The MLVA-31 minimal spanning tree (MST) analysis of *B. anthracis* (Fig. 1) represents 319 strains from southern Africa, where five lineages were denoted by colour. The majority of the investigated strains are predominant in the A.Br.005/006 lineage (pink nodes). The rare B-clade strains are represented by light green nodes found only in Limpopo Province. The A.Br.001/002 lineage (yellow nodes) are present in both KNP and NCP. The non-coloured nodes represent a unique lineage of strains described only in NCP and its associated Transfrontier regions. This cluster belongs to the Ghaap region, NCP that consists of the 1995/8 and 2008/9 that differ by nine up to 17 VNTR loci. The Aust94 (purple nodes) and A.Br.003/004 (olive nodes) lineages are made up of multiple individual nodes unlike the larger nodes constituting clonal genotypes i.e. A.Br.005/006 and B.Br.001/002.

The MLVA-31 minimal spanning tree analysis of *B. anthracis* strains from 1970 to 1990 clustered most of the KNP strains from 1970 to 1980 (Supplementary Fig. 1A, yellow nodes) in the B-clade while most strains from 1990 to 1991 (Supplementary Fig. 1A pink) clustered in the Ancient A-clade. All 1970–1990 KNP isolates in the B-clade including sequenced strains A3, A5, A8, A16, HP8, HP12 (collected from Pafuri soil in 1975) and Z21 (collected from kudu in 1975) are tightly clustered (Supplementary Fig. 1A). The only other B-clade strain outside of this time period is KC2011 collected from a Cheetah outside the endemic region of Limpopo Province, but it groups on its own, belonging to the B.Br.001/002 lineage instead of B.Br.Kruger [5]. Thus, the B-clade strains were divided into two B-clade genotypes by the MST analysis, with certain single genotypes being distinctive on 1–2 VNTRs before 1991 (Supplementary Fig. 1A, yellow and pink nodes) in KNP (Supplementary Fig. 1B, blue-purple nodes). A lower repeat copy number of VNTRs Bams13 and Bams30 was observed among B-clade strains assigned with 6 copies (Supplementary Table 1). The sanger sequencing results for the amplicons matched the copy number. An exception in the copy number of Bams23 for some of the South African isolates was denoted as 10.7 rather than 11 due to a partial repeat unit consistently found in northern KNP.

The A-clade KNP strains grouped in the major (A.Br.005/006) lineage, which consists of also minor lineages (Supplementary Fig. 1A). The most prevalent A.Br.005/006 genotype was different at one to five VNTR loci from the genotypes of outbreaks in 2010–2013. Between 1990 and 1993, the A-clade strains that were only marginally represented in the 1970’s and 1980’s, began to dominate the outbreaks. The largest node belongs to a single/clonal genotype with clear overlap between the outbreak years of 1970 to 1999. All the outbreaks in years thereafter tend toward smaller clonal genotypes restricted temporally, except for 1998, 2008 and 2009 that demonstrate extensive diversity in genotypes. The difference between the dominant genotype in 2012 (light-blue) and the dominant genotype in 2013 (sky-blue) is three loci i.e. Bams15, VrrC1 and VrrA. The KNP anthrax outbreak in 2010 was caused by two dominant genotypes and various single genotypes, while the 2012–2013 outbreak was caused by a dominant genotype each and various single genotypes (Supplementary Fig. 1A). In 1992, A.Br.Aust94 genotype that occurred in NCP also occurred in KNP, and various single A.Br.001/002 (Sterne/Ames) genotypes were identified in 2012 in KNP and NCP (Supplementary Fig. 1B). Of the 319 strains, 11 isolates were missing the plasmid pXO2-related loci pXO2, VNTR16, and VNTR17 (*n* = 4) from 2010 KNP blood smear isolations and seven strains from the OVI NCP archival collections (Supplementary Table 1). It should be noted that these missing loci create a genotypic bias in the MST analysis as missing values will be considered as “zero” alleles, thus contributing unique genotypes. In Supplementary Fig. 1B, it becomes clear that the larger clonal genotypic groups are within the National Parks, specifically KNP (seen in sky blue). The Vaalbos National Park (which no longer exists) had two dominant genotypes in 1992 of which one was unique to the NCP, while the other grouped with the dominant KNP genotype during that period. There is a diversity of genotypes in Mpumalanga, NCP, Rondebosch, and Richtersveld Transfrontier Park from this dataset.

### Whole genome sequencing and placement of the strains

**Fig. 2:**
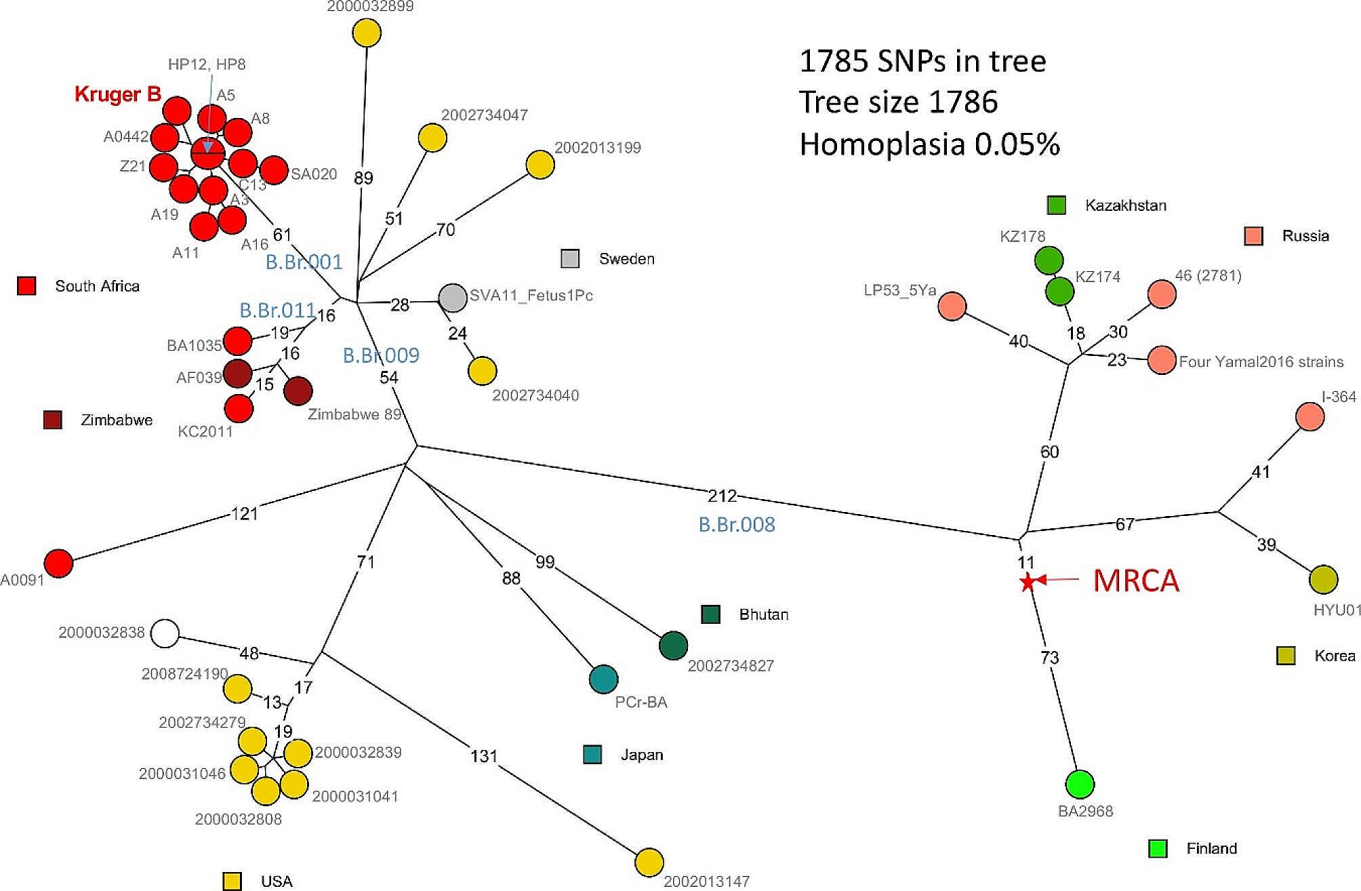
Phylogenetic maximum parsimony tree of the *Bacillus anthracis* strains based on 1785 chromosomal SNPs analysis indicating the clustering of the B.Br.001/002 clade with the ten strains. Nodes are coloured according to geographic origin, and labeled with strain identity. Branch lengths above 9 are indicated in this phylogenetic tree

The short-read sequence data from ten B-clade strains could be assembled in 28 to 72 contigs (Table 2). With the exception of strain A3, which has a 5.29 Mb genome, average genome size is 5.41 Mb. All of *B. anthracis*’ traits were predicted by the genome’s virulence genes, which included the toxin genes (*lef*, *pag*, and *cya*). All the *B. anthracis* plasmid pXO2 was found to include all of the capsular genes (*capDABCE*) in the sequenced genomes. The phylogenetic tree comprised of 70 representative genomes from the A and B-clade. Diverse of the A-clade South African strains are reflected in the A-clade branch with other strains from A.Br.101, A.Br.064, A.Br.001/002 and A.Br.005/006. The phylogenetic tree was defined by 6265 chromosomal SNPs that placed the sequenced B-clade strains (*n* = 10) of KNP with other global B- clade strains from Sweden and Zimbabwe (Fig. 2). A total of 397 SNPs made up the phylogenetic global structure of the B-clade genomes (Fig. 2). The South African KC2011 strain isolated from a cheetah in 2011 shares core SNPs with Zimbabwean ZIM89 strain. In the study, the 1975 KNP strains isolated from soil (*n* = 9) and Z21 (*n* = 1) from the carcass of a kudu are catered by B.Br.001 SNP branch that groups with the South African KrugerB and A0442 strains. No homoplasy was observed among the sequenced genomes showing diverse strains present in Pafuri, KNP.

**Table 2:** Summary statistics for the ten *de novo* assembled *B. anthracis* B- clade draft genomes

*B. anthracis* strains	Sequence coverage (X)	Number of contigs	N50	Genome length	GC %	Plasmids (pXO1 and pXO2)	Predicted coding sequences	Total number of RNA
Ames ancestor	-	3	-	5,503,926	35.1	+	5757	
A3	131.8	36	275,881	5,296,518	35.1	+	5590	18
A11	119.1	27	700,661	5,424,547	35.1	+	5914	55
A5	124.2	31	408,653	5,402,392	35.1	+	5694	26
A16	90.8	38	275,881	5,423,735	35.1	+	5718	13
A8	101.5	72	154,041	5,417,873	35.1	+	5706	58
A19	76.6	34	309,999	5,422,453	35.1	+	5721	15
C13	112.8	28	586,769	5,423,260	35.1	+	5716	58
HP8	73.5	72	154,041	5,417,873	35.1	+	5706	58
HP12	90.2	36	262,421	5,422,775	35.1	+	5721	16
Z21	120.1	41	324,409	5,428,551	35.1	+	5717	29

## Discussion

In this study, we confirmed the decline of the B-clade strains from 1970 to 1991 as no B-clade strains have been reported in KNP since 1992. Furthermore, the genetic diversity of KNP strains is high with most in the Ancient A lineage with evidence of genetic drift that need to be investigated with higher resolution whole genome sequencing. Clustering analysis of strains based on MLVA-31 showed distinctive A.Br.005/006 SNP branch with three identified sub-clusters. Meanwhile the identified B-clade genomes sequenced in this study grouped with KrugerB and A0442 strains previously reported in South Africa. The South African KC2011 (a cheetah from 2011) and A0091 (1939, unknown host) are demarcated by B.Br.011 and B.Br.009, respectively. This work illustrates the dynamics of the *B. anthracis* population in South Africa.

In South Africa, the A-clade strains are distributed in both the enzootic and non-enzootic areas, while the rare B-branch strains are restricted to the enzootic Pafuri region. The Pafuri region has diverse strains [8], as shown in this study with distinctive A-clade *B. anthracis* strains, in addition to the B-clade strains. In the KNP, both the northern and central regions are known to be the foci of anthrax epidemics [8], although the northern region is accepted to be the enzootic area. Geographical distribution showed that the ancient A-clade strains are mainly dominant in the middle of the park, whereas B-clade strains were confined to the northern part of KNP before the 1990s. Since the 1990s, the ancient A-clade strains have been present in the central and northern KNP regions [8]. Since the 1990s, only the rare B-clade KC2011 strain has been isolated from a cheetah sanctuary outside KNP [5].

During the 1970–81 anthrax outbreaks clade B represented approximately a quarter of associated strains [6]. After the 1980s, there were much fewer B-clade strains recorded in the northern section of KNP, with practically all the strains belonging to the ancient A-clade. Possibly due to the diverse range of soil types and distinctive ecology of the pans between the Limpopo and Luvhuvhu Rivers, the B-clade strains are rare and have only been reported in Pafuri, KNP [8]. *B. anthracis* strains from South Africa were previously genotyped using MLVA-12 and found to cluster in the B-clade (B1 clade/Kruger B) [5, 8, 11]. The larger clonal genotypes tend to occur in the north of KNP, which is endemic for anthrax. In endemic Pafuri, anthrax cases occur year-round. Large outbreaks are considered to occur when anthrax cases occur in “non-endemic” areas. These include ranger sections down south to the middle of the KNP. In the past, anthrax outbreaks were common in the dry seasons, with major outbreaks flaring every decade [6]. Since the advent of the new century, anthrax outbreaks have become more commonplace following the rainy season, especially after flooding [27]. The change in these ecological and environmental cues could be responsible for the dominance of the A-clade lineages in recent decades, although this is merely speculative. The difference in isolated diversity between NCP and KNP can be attributed to the restrictions on animal movement and natural resources on the farms in NCP [11, 28, 29]. Each farm serves as its own outbreak site, perpetuating strains within the confines of the farm’s borders. This echoes the findings of Beyer et al. [30, 31], where the greatest genotypic diversity was found outside Etosha National Park. A similar diversity is observed in the conservation areas bordering KNP in Mpumalanga (Supplementary Fig. 1B: turquoise), where animal movement is also restricted.

Furthermore, the four isolates missing plasmid pXO2 from KNP were isolated from old blood smears (unpublished data). Isolates from anthrax cases in the NCP are frequently found to be missing plasmid pXO2 during routine diagnostics [32]. Loss of plasmids is known to occur during storage [33] as is the case with NCP/5, NCP/7 and NCP/9. This finding appears to be common amongst strains obtained from NCP and may also be caused by environmental factors such as the high calcium content of the dolomitic environment of the Ghaap Plateau, which could lead to increased porosity of the bacterium and plasmid curing [5, 28, 32, 34]. The pXO2 plasmid encodes the region responsible for encapsulation and is also needed for virulence [35], therefore plasmid curing must occur after infection and fatality of an animal. It has also been noted that pXO1 could be lost during passage. This results in a genotypic bias during MLVA typing due to the four VNTR loci located on the plasmids.

Monomorphic bacteria can be distinguished using MLVA since the VNTR loci are thought to have high mutation rates [36]. The technique is also considered to have correspondingly high homoplasy and homoplasticity. This is a drawback in assessing a definitive overview of the population structure and evolution of bacteria like *B. anthracis* [20, 37]. Despite this, MLVA typing enabled us to differentiate multiple genotypes from the same carcass, as seen with Roan 5 and Roan 23 from the 2012 anthrax outbreak in Mooiplaas, KNP, and is convenient as a screening tool to select strains most relevant for whole genome sequencing. In this study, ten sequenced genomes were compared with the publicly available genomes and previously sequenced South African genomes [7, 13, 16–18] to determine the placement of the rare B-clade genomes. This study determined the phylogenetic structure of *B. anthracis* in different global lineages and furthermore South African B-clade belongs to B.Br.001, B.Br.009 and B.Br.0011. The sequenced B-clade strains are from the northern section (Pafuri) of KNP, isolated in 1975 and related with South African KrugerB and A0442 strains. The strains KrugerB and A0442 were both isolated in KNP with no specific park location. The sequenced Z21 strain was isolated from a kudu (*Tragelaphus strepsiceros*), while the other sequenced strains were isolated from soil. The KC2011 strain was isolated in 2011 from a cheetah in a private game reserve bordering KNP in Limpopo Province [7] and shows to be closely related with the Zimbabwean ZIM89 strain. They are defined by the B.Br.011 branch with low number of SNPs that are distinctive. The A0091 strain is a South African isolate reported from an anthrax outbreak in 1939 with an unknown source and region defined by B.Br.009 SNP branch. Interestingly only the VNTR bams13 encoding the *BclA* gene was examined, with low repeat copy numbers of the VNTRs recorded on the B-clade KNP strains based on MLVA-31. This observation might reflect an influence on the molecular mass of the proteins synthesized by different *B. anthracis* strains [38]. However, the genes that play a role in the survival of A-clade strains over B-clade strains are still not clearly understood. The protein content of the *BclA* between A-clade and B-clade strains could be further exploited on the South African genomes as a low-copy number of repeat units determined in the B-clade strains was observed in this study. This might reflect the differences in the spore-coat region with less protein content that could influence the survivability of B-clade strains.

## Conclusions

Ultimately a well-defined phylogenetic structure was determined which placed the rare B-clade genomes into a distinctive sub-branch in KNP. The MLVA-31 data showed the existence of the clonal A-clade strains that is predominant in KNP, while the NCP strains form a distinctive sub-clade. The rare B-clade constituting the B.Br.009 clade is only represented in KNP and is undetected since the early 1990’s. The B-clade showed variety of strains represented by low numbers of SNPs. Therefore, an in-depth mining of complete genomes of B-clade strains is needed to expand our understanding of the disappearance of the rare B-clade clade coupled by well-designed experiments for spore survivability in different soil environments. This study established a baseline foundation for future epidemiological studies of anthrax by exploiting the use of high resolution wgSNP analysis supported by MLVA-31 data for genotyping of *B. anthracis* strains.

### Electronic supplementary material

Below is the link to the electronic supplementary material.

Supplementary Material 1

Supplementary Material 2

Supplementary Material 3

Supplementary Material 4

## Data Availability

This publication contains all of the MLVA-31 allele profiles of the 319 strains that were examined. The sequenced *B. anthracis* strains’ nucleotide accession numbers, which have been uploaded to the sequence reads archive database under the bioproject PRJNA642997, are provided in this work.
