# Supplementary material for: Bacillus anthracis in South Africa, 1975–2013: are some lineages vanishing?
Source: BMC Genomics. 2024 Jul 30;25:742. doi: 10.1186/s12864-024-10631-5 (PMC11290001; doi:10.1186/s12864-024-10631-5)
Supplement: Supplementary file 3 — Supplementary Material 3 [file 12864_2024_10631_MOESM3_ESM.docx]

Supplementary Table 3. Nucleotide accession numbers of the sequenced *B. anthracis* strains (n =10) from Pafuri, Kruger National Park used in this study.

| Accession | Study | Bioproject accession | Biosample accession | Sample name |
| --- | --- | --- | --- | --- |
| SRR12115353 | SRP269316 | PRJNA642997 | SAMN15402701 | A3 |
| SRR12115352 | SRP269316 | PRJNA642997 | SAMN15402702 | A5 |
| SRR12115349 | SRP269316 | PRJNA642997 | SAMN15402703 | A8 |
| SRR12115347 | SRP269316 | PRJNA642997 | SAMN15402705 | A16 |
| SRR12115346 | SRP269316 | PRJNA642997 | SAMN15402706 | A19 |
| SRR12115345 | SRP269316 | PRJNA642997 | SAMN15402707 | C13 |
| SRR12115344 | SRP269316 | PRJNA642997 | SAMN15402708 | HP8 |
| SRR12115343 | SRP269316 | PRJNA642997 | SAMN15402709 | HP12 |
| SRR12115342 | SRP269316 | PRJNA642997 | SAMN15402710 | Z21 |
| SRR12115348 | SRP269316 | PRJNA642997 | SAMN15402704 | A11 |
