# Supplementary figures and images for: Bacillus anthracis in South Africa, 1975–2013: are some lineages vanishing?
Source: BMC Genomics. 2024 Jul 30;25:742. doi: 10.1186/s12864-024-10631-5 (PMC11290001; doi:10.1186/s12864-024-10631-5)

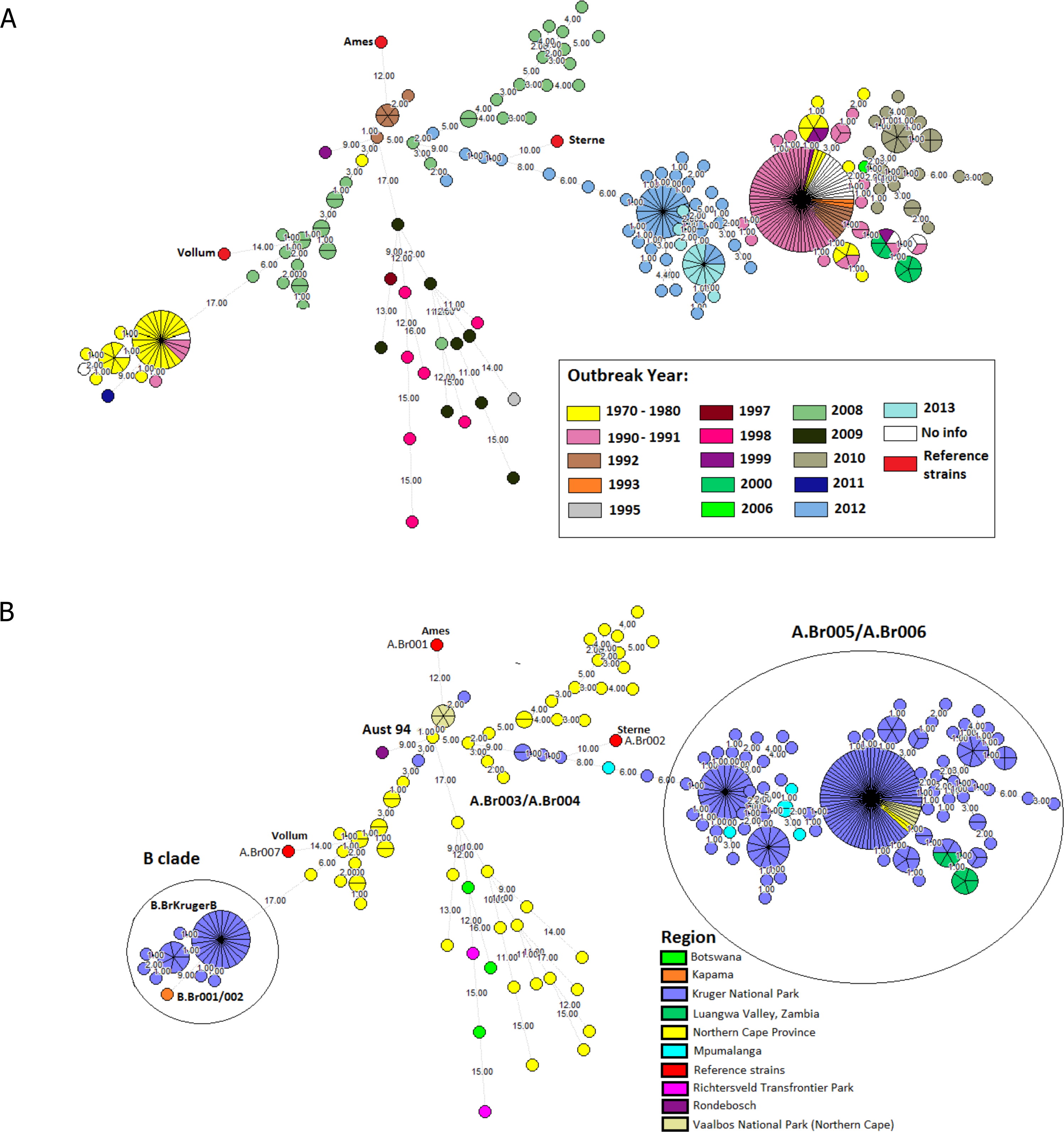

Supplement: Supplementary file 4 — Supplementary Material 4 [file 12864_2024_10631_MOESM4_ESM.tiff]
